# Alcoholic Liver Disease: Role of Cytokines

**DOI:** 10.3390/biom5032023

**Published:** 2015-08-28

**Authors:** Manuela G. Neuman, Yaakov Maor, Radu M. Nanau, Ehud Melzer, Haim Mell, Mihai Opris, Lawrence Cohen, Stephen Malnick

**Affiliations:** 1 *In Vitro* Drug Safety and Biotechnology, University of Toronto, Toronto, ON M5G 0A3, Canada; E-Mails: radu.nanau@utoronto.ca (R.M.N.); M_neuman@rogers.com (M.O.); 2 Department of Pharmacology and Toxicology, Faculty of Medicine, University of Toronto, Toronto, ON M5G 0A3, Canada; 3 Division of Gastroenterology, Kaplan Health Sciences Centre, Department of Medicine, Faculty of Medicine, Hebrew University, Rehovot 76100, Israel; E-Mails: halishy@netvision.net.il (Y.M.); ehudm@clalit.org.il (E.M.); stephen@malnick.net (S.M.); 4 Israel Anti-Drug Authority, Jerusalem 91039, Israel; E-Mail: haimm@antidrugs.gov.il; 5 Casa de Ajutor Reciproc, Bucharest 031621, Romania; 6 Sunnybrook Health Sciences Centre and Department of Internal Medicine, University of Toronto, Toronto, ON M5G 0A3, Canada; E-Mail: lawrence.cohen@sunnybrook.ca

**Keywords:** alcoholic liver disease, hepatocellular carcinoma, lipopolysaccharide, transforming growth factor, Toll-like receptor, tumor necrosis factor

## Abstract

The present review spans a broad spectrum of topics dealing with alcoholic liver disease (ALD), including clinical and translational research. It focuses on the role of the immune system and the signaling pathways of cytokines in the pathogenesis of ALD. An additional factor that contributes to the pathogenesis of ALD is lipopolysaccharide (LPS), which plays a central role in the induction of steatosis, inflammation, and fibrosis in the liver. LPS derived from the intestinal microbiota enters the portal circulation, and is recognized by macrophages (Kupffer cells) and hepatocytes. In individuals with ALD, excessive levels of LPS in the liver affect immune, parenchymal, and non-immune cells, which in turn release various inflammatory cytokines and recruit neutrophils and other inflammatory cells. In this review, we elucidate the mechanisms by which alcohol contributes to the activation of Kupffer cells and the inflammatory cascade. The role of the stellate cells in fibrogenesis is also discussed.

## 1. Introduction

The clinical spectrum of alcoholic liver disease (ALD) includes the accumulation of lipids (steatosis), in the presence or absence of inflammation (steatohepatitis), leading to cirrhosis and an increased risk of hepatocellular carcinoma (HCC) [1]. The mechanism of alcohol-induced hepatotoxicity comprises interactions between the direct toxic effects of alcohol and its metabolites on various cell types in the liver, induction of reactive oxygen species, up-regulation of the inflammatory cascade, as well as other cell-specific effects in the liver [2,3,4]. The histological stages of ALD include fatty liver or simple steatosis, alcoholic hepatitis (AH), and chronic hepatitis that may also be associated with the presence of Mallory’s hyaline, megamitochondria, or perivenular and perisinusoidal fibrosis [5,6]. Fatty liver develops in approximately 90% of individuals who drink more than 60 g alcohol/day. This condition is completely reversible after 4–6 weeks of abstinence, even if fibrosis has already developed [1,7].

The most worrisome feature in liver disease is hepatic fibrosis, which represents the liver’s wound healing response to alcohol-induced injury. Hepatic fibrosis is characterized by the accumulation of interstitial matrix [8,9]. Liver fibrosis results from the activation of hepatic stellate cells. Stellate cells activation is a phenotype transition whose effect is the formation of collagen deposits. Features of stellate cell activation include increased cell accumulation, increased matrix production, enhanced contractility, diminished degradation of the extracellular matrix, release of pro-fibrogenic cytokines, and loss of cellular vitamin A. Alcohol may enhance fibrogenesis through stimulation of stellate cells, generation of lipid peroxides from damaged hepatocytes, metabolic production of toxic acetaldehyde that induces direct fibrogenic activity, and through the activation of Kupffer cells or resident macrophages [10].

## 2. Alcohol and Cytokines

Cytokines are inflammatory mediators that serve important roles in the pathogenesis of many acute and chronic diseases, including alcoholic liver disease [2,3,4]. A wide range of cytokines were assessed in samples of ALD patients. These include tumor necrosis factor (TNF)-α, various interleukins (IL) such as IL-1β, IL-4, IL-6, IL-10, and IL-12, and interferon (IFN)-γ, as well as high-sensitivity C-reactive protein (hsCRP), as described in Table 1 [11,12,13,14,15,16,17,18,19,20,21]. Several studies have uncovered relationships between cytokine levels and the severity of the disease, as well as the presence of co-morbidities. Mean IL-6, IL-8, hsCRP, and TNF-α levels were higher among individuals with more advanced liver disease [16,17]. TNF-α and IL-6 levels could be used to differentiate between patients with compensated cirrhosis and patients with de-compensated alcoholic cirrhosis [21]. Elevated levels of soluble TNF-receptor 55 and soluble IL-2 receptor were associated with bone resorption (deoxypyridinoline urinary excretion) and osteoporosis in patients with alcohol-induced cirrhosis [18]. Elevated IL-6 [hazard ratio (HR) 2.94, 95% confidence interval (CI) 1.69–5.12, *p* < 0.01) and CRP (HR 1.87, 95% CI 1.11–3.15, *p* = 0.02) levels were associated with mortality in a large cohort of human immunodeficiency virus-infected adults with alcohol problems [12].

Table 1 summarizes levels of pro-inflammatory markers in ALD patients [11,12,13,14,15,16,17,18,19,20,21].

**Table 1:** Cytokine and pro-inflammatory markers levels in alcoholic liver disease patients. AH: alcoholic hepatitis; ALD: alcoholic liver disease; CSF: cerebrospinal fluid; hsCRP: high-sensitivity C-reactive protein; IFN: interferon; IL: interleukin; IQR: interquartile range; MCP: monocyte chemoattractant protein; sIL-2R: soluble IL-2 receptor; sTNF-R55: soluble tumor necrosis factor receptor 55; TGF: transforming growth factor; TNF: tumor necrosis factor; VEGF: vascular endothelial factor.

Refs.	Population/Matrix	Findings: Mean ± Standard Deviation or Median (IQR);
[11]	28 alcoholics, 13 controls/CFS	MCP-1 (pg/mL): Healthy controls 425.7; Alcoholics 455.9 (*p* = 0.001) IL-1β: no difference
[12]	400 human immunodeficiency virus-infected adults with alcohol problems followed prospectively/serum	IL-6 (pg/mL): 2.75 (IQR 1.52–4.81) IL-10 (pg/mL): 5.03 (IQR 3.08–8.20) TNF-α (pg/mL): 7.15 (IQR 4.85–9.70) CRP (mg/mL): 1.48 (IQR 0.60–3.47) Cystatin-C (ng/mL): 0.77 (IQR 0.67–0.91) MCP-1 (pg/mL): 577 (IQR 397–815) Amyloid A (mg/mL): 3.2 (1.8–6.4)
[13]	50 cirrhotic patients (60% of alcoholic etiology)/serum	CRP (mg/dL): 0.53 ± 0.21 Lipopolysaccharide-binding protein (ng/mL): 8669 ± 1207 IL-6 (pg/mL): 6.94 ± 3.43
[14]	47 ALD (31 non-cirrhotic and 16 cirrhotic), 18 controls; in serum	TNF-α (pg/mL): Controls 6.12 ± 1.87; ALD Non-cirrhotic: 5.013 ± 2.10; ALD Cirrhotic: 7.18 ± 3.51 IL-4 (ng/mL): Controls 4.62 ± 6.17; ALD Non-cirrhotic: 0.82 ± 1.53; ALD Cirrhotic: 1.89 ± 4.57 IL-6 (pg/mL): Controls 5.94 ± 1.68; ALD Non-cirrhotic: 6.46 ± 4.87; ALD Cirrhotic: 7.79 ± 9.11 IL-10 (pg/mL): Controls 9.00 ± 12.10; ALD Non-cirrhotic: 11.67 ± 24.92; ALD Cirrhotic: 11.71 ± 8.21 IL-13 (pg/mL): Controls 4.06 ± 6.60; ALD Non-cirrhotic: 10.67 ± 19.90; ALD Cirrhotic: 26.42 ± 32.49 IFN-γ (pg/mL): Controls 0.7 ± 0.51; ALD Non-cirrhotic: 6.75 ± 7.64; ALD Cirrhotic: 4.23 ± 2.58
[15]	56 AH patients, 18 age- and sex-matched controls/serum	CRP (mg/mL): Controls 0.36 ± 1.11; AH day 0: 3.68 ± 6.09 (*p* < 0.001); AH day 15: 1.34 ± 1.87 (*p* < 0.001) IL-8 (pg/mL): Controls 6.75 ± 1.79; AH day 0: 57.80 ± 97.05 (*p* < 0.005 *vs.* control); AH day 15: 26.60 ± 35.15 (*p* < 0.005 *vs.* control); IL-10 (pmol/L): Controls 8.22 ± 10.69; AH day 0: 5.13 ± 0.59 (*p* < 0.001 *vs.* control); AH day 15: 5.90 ± 4.10 (*p* < 0.001 *vs.* control) IL-4 (ng/mL): Controls 2.28 ± 1.72; AH day 0: 6.88 ± 10.20 (*p* < 0.05 *vs.* control); AH day 15: 27.49 ± 42.90 (*p* < 0.05 *vs.* control) IFN-γ (pg/mL): Controls 0.66 ± 0.50; AH day 0: 4.74 ± 12.60 (*p* < 0.05 *vs.* control); AH day 15: 2.94 ± 3.68 (*p* < 0.05 *vs.* control) TNF-α (pg/mL): Controls 5.83 ± 1.77; AH day 0: 7.22 ± 7.21; AH day 15: 6.97 ± 3.84 IL-6 (pg/mL): Controls 5.77 ± 1.40; AH day 0: 39.82 ± 81.85; AH day 15: 11.87 ± 21.38 Malondialdehyde: Controls 1.31 ± 0.69; AH day 0: 8.34 ± 6.76 (*p* < 0.001 *vs.* control); AH day 15: 7.16 ± 5.38 (*p* < 0.001 *vs.* control)
[16]	45 stable patients with alcoholic cirrhosis (16 with Child A stage, 19 with Child B stage and 10 with Child C stage), 12 healthy controls; in serum	MCP-1 (pg/mL): Controls 460 (IQR 376–617); Child A stage: 305 (IQR 139–365); Child B: 220 (IQR 169–277) (p<0.05 *vs.* controls and Child A); Child C: 174 (IQR 99–271) (*p* < 0.05 *vs.* controls and Child A) TNF-α (pg/mL): Controls 3.9 (IQR 3.5–6.5) ); Child A stage: 5.6 (IQR 3.4–27.8); Child B: 5.0 (IQR 2.8–33.2); Child C: 7.2 (IQR 4.2–16.8) IL-6 (pg/mL): Controls 24.2 (IQR 1.2–43.1); Child A stage: 23.1 (IQR 8.9–78.2); Child B: 30.3 (IQR 12.4–95.6); Child C: 35.8 (IQR 22.8–85.6) IL-8 (pg/mL): Controls 18 (IQR 8–36) ); Child A stage: 50.3 (IQR 21.2–77.9); Child B: 49.0 (IQR 14.3–80.2); Child C: 94.8 (IQR 46.2–261.4) VEGF (pg/mL): Controls 265 (IQR 96–740) ); Child A stage: 732 (IQR 386–846); Child B: 405 (IQR 97–716); Child C: 429 (IQR 249–599) hsCRP (mg/L): Controls 1.6 (IQR 1.1–5.1) ); Child A stage: 3.6 (IQR 2.1–8.9); Child B: 5.0 (IQR 1.4–7.0); Child C: 8.2 (IQR 7.3–12.8) (*p* < 0.05 *vs.* controls and Child B)
[17]	24 AH patients (5 severe and 19 non-severe disease), 20 healthy controls/plasma	IL-6 (pg/mL): Controls <7.8; Severe AH: 504 ± 681; Non-severe AH: 25 ± 32 (*p* < 0.001 *vs.* severe AH) IL-8 (pg/mL): Controls <15.6; Severe AH: 216 ± 304; Non-severe AH: 37 ± 77 (*p* < 0.05 *vs.* severe AH) TNF-α (pg/mL): Controls <15.6; ; Severe AH: 29 ± 18; Non-severe AH:17 ± 6 (*p* < 0.005 *vs.* severe AH)
[18]	33 ALD cirrhosis, 24 healthy controls/serum	sTNF-R55 (pg/mL): Controls 1.28 ± 0.12; ALD: 5.94 ± 0.48 (*p* < 0.001); sIL-2R (pg/mL): Controls 545.33 ± 37.30; ALD: 953.06 ± 85.03 (*p* < 0.001)
[19]	94 ALD (17 steatosis, 37 AH; 40 cirrhosis), 35 abstainers	IL-12 pg/mL: Controls 39.3 ± 8.3; Steatosis: 74.4 ± 26.2; AH: 163.1 ± 57.8
[20]	24 alcoholic patients; hepatic mRNA expression	TNF-α: fibrosis (F3/F4) 1.74 ± 0.27; (F0/F1):1 ± 0.16 (*p* = 0.039) TGF-β: fibrosis (F3/F4) 2.33 ± 0.17; (F0/F1):1 ± 0.16 (*p* = 0.0001) Fas ligand: fibrosis (F3/F4) 1.55 ± 0.13; (F0/F1); 1.55 ± 0.13 (*p* = 0.007)
[21]	43 patients with ALD cirrhosis (29 compensated and 14 de-compensated), 30 healthy controls; in plasma	TNF-α (pg/mL): Controls 46 ± 10.0; cirrhosis 30.2 ± 2.10 (*p* < 0.05 *vs.* control); De-compensated cirrhosis; 24.5 ± 1.9 (*p* < 0.05 *vs.* control and *vs.* compensated) IL-6 (pg/mL): Controls 33.11 ± 2.06; cirrhosis 41.06 ± 4.57 (*p* < 0.05 *vs.* control): De-compensated cirrhosis 53.5 ± 4.8 (*p* < 0.05 *vs.* control and *vs.* compensated) CRP (mg/L): Controls 6.99 ± 0.98; cirrhosis: 20.07 ± 13.90 (*p* < 0.05 *vs.* control); De-compensated cirrhosis: 24.9 ± 15.0 (*p* < 0.05 *vs.* control)

Fibrosis deposition is also influenced by the dysfunction of the wound-healing process in the liver [22]. A large sample of 260 AH and 1180 hepatitis C virus (HCV) patients revealed that TNF-α levels were higher in ALD compared to HCV (*p* < 0.05). Transforming growth factor (TGF)-β levels were elevated in patients with more advanced fibrosis, regardless of the etiology of the liver disease [23]. A recent study compared the cytokines profile of 30 patients with alcoholic cirrhosis and 15 patients with HCV cirrhosis. IL-12, IL-6 and TNF-α levels were higher in alcoholic cirrhosis (*p* < 0.05 for all), while IL-1β and IL-10 levels were comparable between patients with alcoholic or HCV cirrhosis. TNF-α levels were elevated in ALD patients with cirrhosis compared to ALD patients without cirrhosis (*p* < 0.05). TNF-α levels were further elevated in ALD patients compared to non-alcoholic fatty liver disease (NAFLD) patients (*p* < 0.0001) [24].

Changes in cytokine levels were further analyzed in individuals undergoing alcohol withdrawal. Parameters of alcoholism, such as the pro-inflammatory cytokine IL-6, were found to decrease from baseline through day 15 in a small sample of 52 alcohol-dependent individuals without liver diseases undergoing alcohol detoxification [25].

Hepatic TGF-β (*p* < 0.001), TNF-α (*p* = 0.034) and Fas ligand (*p* = 0.008) genes expression was correlated with fibrosis in a small sample of alcoholic patients [20]. Single-nucleotide polymorphisms in *IL-1*β (−511C/T), *IL-10* (−592C/A), *TNF-*α (−308G/A, −238G/A), *TGF-*β*1* (−509C/T) and *CTLA4* (+49A/G) were assessed in a South Indian population of 181 patients with alcoholic cirrhosis and 110 controls. Single-nucleotide polymorphisms associated with increased risk of ALD include the −238AA *TNF-*α variant [odds ratio (OR) 2.36, 95% CI 1.1–5.3], −511C *IL-1*β allele (OR 2.64, 95% CI 1.2–5.9), −509C *TGF-*β*1* allele (OR 4.71, 95% CI 1.7–13.1), and −592A *IL-10* allele (OR 2.17, 95% CI 1.1–4.8) [26]. There were no differences between genetic polymorphism at codon 10 in the *TGF-*β gene and the development of alcoholic liver cirrhosis in a Korean population [27].

## 3. Alcohol and Chemokines

IL-8, a chemokine produced by a variety of cells including monocytes, macrophages, Kupffer cells and hepatocytes, can activate neutrophils. Peripheral neutrophilia and liver neutrophil infiltration are frequently noted in patients with ALD [2,28]. Patients with high serum IL-8 have a higher mortality rate than those with lower levels. Serum IL-8 levels decreased gradually with abstinence from alcohol [3].

A recent review by Gao and Xu [29] discusses several chemokines believed to play important roles in AH. These include several members of the CXC subfamily such as IL-8, Gro-α, CXCL5, CXCL6, CXCL10, and platelet factor 4, whose levels were significantly elevated in AH livers compared with normal healthy control livers. Other molecules believed to play a role in AH include the pro-inflammatory cytokine IL-17 and the chemokines CXCL9 and CXCR3 [30]. Levels of the CC chemokine CCL2 (monocyte chemoattractant protein (MCP)-1), but not CCL5, were also elevated. Higher expression levels of IL-8, CXCL5, Gro-γ and CXCL6 were associated with a less favorable prognosis in AH patients [31]. Many CXC and CC family members were markedly elevated in AH biopsy samples compared with healthy control liver samples in another study [32]. The levels of chemokines correlate with neutrophil infiltration and the severity of portal hypertension [31].

The hepatic and serum levels of CCL20 are also increased in AH. The interaction between CCL20 and CCR6 regulates liver inflammation, fibrosis and tumorigenesis [33]. In AH patients, CCL20 mRNA expression and serum levels positively correlated with one another, suggesting that the liver may be an important source of CCL20 [33]. A strong correlation was further observed between serum CCL20 and lipopolysaccharide (LPS) levels, the main inducer of this chemokine. Increased hepatic CCL20 expression and serum CCL20 levels correlated with short-term mortality (within 90 days of hospitalization) in AH patients [33].

Levels of pro-inflammatory factors (IL-6, CCL2, CCL5, IL-8, osteopontin, semaphorin 7A), inflammasome components (IL-1β, IL-18, caspase-1), the total macrophage marker CD68 and fibrosis markers (TGF-β1) were increased in liver tissue samples in ALD patients with severe liver damage compared to those with mild liver damage. A positive correlation was found between the mRNA expression of chemokines involved in the recruitment of immune cells such as osteopontin (*p* < 0.05), semaphorin 7A (*p* < 0.001) and IL-8 (*p* = 0.06), and severe steatosis (≥33%) [34]. Alcohol withdrawal led to an increase in CCL18 expression (*p* = 0.001) in subcutaneous adipose tissue. Additionally, alcohol withdrawal was associated with decreased expression of the pro-inflammatory markers IL-18, CCL2, osteopontin and semaphorin 7A, and of the macrophage marker CD68 in subcutaneous adipose tissue by 1 week in patients with mild ALD, with an increase in the M2 marker CCL18 [34].

Higher serum osteopontin levels were observed in patients with more advanced hepatic inflammation (*p* = 0.047) and more advanced hepatic fibrosis (*p* < 0.001) in a sample of 204 heavy drinkers. Hepatic osteopontin expression correlated well with serum osteopontin levels, as well as with hepatic inflammation, fibrosis, TGF-β expression and neutrophils accumulation among 72 heavy drinkers [35]. The hepatic expression of osteopontin was found to be greatly elevated among patients with AH compared to patients with hepatic diseases of other etiology or with healthy controls. Hepatic mRNA expression and serum osteopontin levels correlated with one another in AH patients, and with liver damage [36]. The presence of the CXCL1 rs4047 A allele was higher among patients with alcoholic cirrhosis (65.3%) than among alcoholic controls (54.8%) (OR 1.55 95% CI 1.025–2.350 *p* = 0.04) in a large sample of 458 patients with alcoholic cirrhosis (170 with HCC), 115 alcoholics without liver disease and 342 healthy controls. This allele was not associated with HCC development [37]. CXCL1 expression was further assessed among individuals carrying the CXCL1 rs4074 G/A polymorphism in a subsample of patients with high alcohol consumption (>300 g/week). The homozygous GG genotype in alcoholic patients was associated with CXCL1 expression similar to that of healthy controls. On the other hand, the presence of the A allele among patients with alcoholic cirrhosis was associated with significantly higher serum CXCL1 levels [GA = 212.7 ± 16.5 (*p* = 0.034) and AA = 287.9 ± 73.3 pg/mL (*p* = 0.028) compared to the GG genotype, and GA = 133.7 ± 10.5 (*p* = 0.003) and AA = 152.4 ± 13.4 pg/mL (*p* = 0.03) compared to the corresponding genotypes in healthy controls] [37].

Hepatic gene expression profiling assessed by DNA microarray revealed that the expression of 207 genes was altered between 15 patients with severe AH (ABIC score ≥ 6.71 points) and seven normal controls [32]. Some CC chemokines, particularly MCP-1, were up-regulated in AH patients compared to controls, while the expression profiles of others was similar. Some TNF superfamily receptors such as FAS, TRAILR1 and Fn14 were over-expressed in AH, while there was no difference in the expression profiles of TNF superfamily ligands such as TNF-α and Fas ligand between AH patients and controls. Hepatic gene expression of Fn14 (HR 1.05, 95% CI 1.00–1.11, *p* = 0.03), IL-8 (HR 1.14, 95% CI 1.02–1.26, *p* = 0.019), CXCL-5 (HR 1.01, 95% CI 1.004–1.02, *p* = 0.006), CXCL1 (HR 1.001, 95% 1.00–1.003, *p* = 0.018), and CXCL6 (HR 1.01, 95% CI 1.004–1.03, *p* = 0.009) predicted 90 day mortality in AH patients. Higher Fn14 hepatic expression predicted poorer 90-day survival rates. High Fn14 gene expression (>22-fold-expression) also predicted more severe portal hypertension (*p* = 0.04). The Fn14 gene expression profile was correlated with the ABIC score, an indicator of AH disease severity (*p* = 0.01). Fn14 protein expression was identified primarily in parenchymal cells around fibrogenic areas in AH patients. It was expressed in hepatocytes at the edge of regenerative nodules [32]. Higher mRNA expression of the M2 markers (CD206 and CD163) predicted limited hepatic injury in liver biopsies in alcohol drinkers. On the other hand, expression of IL-10 and of M1 markers (TNF-α) predicted more severe hepatic injury [38].

Resistin was markedly increased in AH patients compared with cirrhotic controls in a sample of 76 patients with severe acute AH and 25 patients with alcoholic cirrhosis used as controls included in a prospective, case-control study. Elevated resistin levels predicted decreased survival rate. Platelet activation inhibitor-1 expression was mildly enhanced in AH subjects, while serum leptin levels were decreased in AH patients. Adiponectin and adipsin levels were similar between groups. Neither of platelet activation inhibitor-1, leptin, adiponectin or adipsin could be used to predict the 180-day survival rate. In contrast, all of TNF-α, IL-6, IL-8, and IL-15 were significantly increased in patients with AH when compared to cirrhotic subjects, and all could be used to predict decreased survival rate [39].

## 4. Alcoholic Hepatitis and Lipopolysaccharides

Liver inflammation is collectively described by the elevated expression of pro-inflammatory genes, which is common to all liver conditions collectively referred to as hepatitis. Several central events are important in ALD, including bacterial translocation (the migration of bacteria and bacterial parts from the intestine to extra-intestinal organs), Toll-like receptor (TLR) stimulation by bacterial ligands, as well as intestinal bacterial overgrowth and dysbiosis [40]. Alcohol has been suggested to also alter the intestinal microbiota, and a correlation between dysbiosis and endotoxemia is believed to exist [41,42].

Alcohol consumption is a known cause of increased gut permeability, facilitating the translocation of gut microbiota into the circulation. In turn, this leads to increased liver exposure to LPS, which causes liver injury via TLR4 activation [42]. Human TLR4 recognizes LPS via the co-receptors CD14 or MD-2. This recruits MyD88, forming a TLR4-MyD88 complex. This complex further activates nuclear factor NF-κB. This phenomenon leads to increased production of pro-inflammatory cytokines, such as TNF-α, IL-6, and IL-1β [43,44].

Chronic alcohol consumption increases gut permeability, permitting the translocation of LPS from the intestinal lumen to the portal circulation. During AH, LPS interacts with host immune cells and triggers the release of pro-inflammatory cytokines. TLRs are important components of the innate immune system and recognize pathogenic toxins. TLR4 is particularly important in AH. Ultimately, the activation of this pathway leads to the recruitment of inflammatory cells [45]. Importantly, intestinal inflammation can result in disruption of epithelial tight junctions and bacterial translocation, and this, too, can play a significant role in ALD development [46,47]. A positive association is known to exist between the circulating levels of bacterial products in plasma and the severity of liver disease in patients, particularly liver disease of alcoholic etiology [40].

Increasing serum LPS levels paralleled increasing blood alcohol levels. Microbial translocation from the gut was shown through a significant increase in serum cytokine levels. This was observed through 24 h. Significant induction of inflammatory cytokines such as TNF-α (*p* < 0.05), IL-6 (*p* < 0.05) and MCP-1 (*p* < 0.05) ensued following treatment of whole blood samples with LPS levels comparable to those observed in healthy volunteers after binge drinking [10].

Elevated LPS levels were noted in patients with severe AH in one study, and this was closely related to elevated levels of pro-inflammatory cytokines [17,48]. LPS levels were comparable between alcoholics with or without liver disease, and they were significantly higher in both patient groups compared to healthy subjects (*p* = 0.001). Dysbiosis was observed with a higher incidence among alcoholics than among healthy subjects [49]. Markwick *et al.* [50] further observed impaired antibacterial innate and adaptive immune responses in patients with acute AH compared to patients with stable advanced alcohol-related cirrhosis or healthy controls.

Bacterial translocation is initiated when the intestinal epithelium gets damaged. For example, acetaldehyde, an alcohol metabolite, has the potential to damage the intestinal epithelium. In turn, bacterial products (pathogen-associated molecular patterns, which recognize conserved features of microbial products) such as LPS, flagellin, peptidoglycan, and bacterial DNA can be measured in portal circulation. Plasma LPS levels are elevated in chronic drinkers. The innate immune system recognizes bacterial products, and the result of the interaction between TLR4 and its agonist, LPS, is the production of pro-inflammatory cytokines and chemokines [40,42].

In a study conducted by Almeida and colleagues [51] there were lower levels of peripheral blood CD4^+^ CD25^hi^ CD127^−/10^ circulating regulatory T cells in AH patients than in chronic alcoholic patients without liver disease or healthy controls. T-cell proliferation rates were higher in AH. LPS-binding protein (LBP) levels were elevated in AH patients compared to controls (median 6.52 μg/mL *vs.* median 2.47 μg/mL, *p* < 0.005). Systemic inflammatory response syndrome was associated with elevated plasma LBP levels in AH patients. Increased secretion of pro-inflammatory cytokines was observed in AH patients despite a normal numbers of peripheral blood monocytes and all dendritic cells subsets.

A prospective study in 50 AH patients and 46 controls (26 patients with stable alcoholic cirrhosis and 20 healthy individuals) followed for 30 days measured plasma levels of soluble CD163 and liver tissue expression of CD163 as a specific marker of inflammatory macrophage activation, and plasma LPS, sCD14, and LBP as key components of the LPS pathway. Plasma sCD163 levels were elevated in AH patients (mean 15.4 mg/L) compared to alcoholic cirrhosis (mean 11.5 mg/L; *p* < 0.002) and healthy individuals (mean 1.5 mg/L; *p* < 0.001). LPS was detectable in peripheral venous blood in 48.0% of AH patients and only 15.0% of healthy controls. Plasma LBP was higher in AH patients (mean 29 μg/mL) than in alcoholic cirrhosis (mean 17 μg/mL; *p* < 0.002) and healthy controls (mean 15 μg/mL; *p* < 0.002). Similarly, sCD14 was higher in AH patients (mean 2.4 μg/mL) than in healthy controls (mean 1.3 μg/mL; *p* < 0.0001), but it was similar in alcoholic cirrhosis (mean 2.4 μg/mL). All of LPS, LBP, and sCD14 were shown to decline during the 30 day follow-up. These parameters did not predict the risk of infection. In contrast, higher sCD163 at study entry predicted mortality in AH patients (mean 21 mg/L among patients who died and mean 15 mg/L among patients who lived, *p* < 0.001) [52].

## 5. Conclusions

Clinical and pathological features of ALD that are mediated by cytokines, include cachexia, cholestasis, fibrosis, synthesis of acute-phase proteins, and hypergammaglobulinemia. Pro-inflammatory cytokines such as TNF-α and IL-6 are mainly involved in cholestasis and the synthesis of acute-phase proteins. The pro-fibrogenic cytokine TGF-β, which is released by activated Kupffer cells and stellate cells, is the critical cytokine involved in fibrosis. In patients with progressive ALD, the balance between pro-inflammatory and anti-inflammatory cytokines may be shifted toward the pro-inflammatory axis, thus the counteracting anti-inflammatory cytokines are unable to control inflammation and fibrosis.

## Abbreviations

ALD: alcoholic liver disease
AH: alcoholic hepatitis
CI: confidence interval
HCC: hepatocellular carcinoma
HCV: hepatitis C virus
HR: hazard ratio
hsCRP: high-sensitivity C-reactive protein
IL: interleukin
IFN: interferon
LBP: lipopolysaccharide-binding protein
LPS: lipopolysaccharide
OR: odds ratio
TGF: transforming growth factor
TLR: Toll-like receptor
TNF: tumor necrosis factor
